# Evaluation of the Systemic Immune-Inflammation Index, Neutrophil-to-Lymphocyte Ratio, and Platelet-to-Lymphocyte Ratio in Patients Diagnosed with Lung Cancer by Bronchoscopy in Relation to Disease Stage and PET/CT SUVmax

**DOI:** 10.1055/s-0046-1827247

**Published:** 2026-08-05

**Authors:** İlknur Kaya, Nuray Kurt, Ümran Erbay, Şebnem E. Parspur, Feride Marım, Mehmet Doğan, Şerife Kaya

**Affiliations:** 1 Department of Chest Diseases, Faculty of Medicine, Kütahya Health Sciences University, Kütahya, Türkiye

**Keywords:** lung cancer, ^18^ F-FDG PET SUVmax, systemic immune-inflammatory index

## Abstract

**Introduction:**

Systemic inflammation in lung cancer is closely associated with tumor progression and prognosis. Hematological parameters such as the systemic immune-inflammation index (SII), neutrophil-to-lymphocyte ratio (NLR), and platelet-to-lymphocyte ratio (PLR) are readily available, low-cost biomarkers. This study aimed to evaluate the associations of SII, NLR, and PLR with disease stage and the maximum standardized uptake value (SUVmax) measured by ^18^ F-fluorodeoxyglucose positron emission tomography/computed tomography ( ^18^ F-FDG PET/CT) in patients with lung cancer diagnosed by bronchoscopy.

**Materials and Methods:**

This retrospective study included 184 of 357 patients who underwent fiberoptic bronchoscopy at the Department of Respiratory Medicine, Kütahya Health Sciences University, between January 2023 and March 2025 and had complete staging data. Demographic characteristics, histopathological diagnoses, ^18^ F-FDG PET/CT SUVmax values, and SII, NLR, and PLR values calculated from complete blood count parameters were recorded.

**Results:**

A total of 184 patients were included; 172 (93.5%) were male and 12 (6.5%) were female, and the mean age was 67.9 ± 8.4 years. The most common histopathological diagnosis was non-small cell lung cancer (NSCLC), accounting for 84.8% of cases. No significant associations were found between SII, NLR, or PLR and disease stage or SUVmax. NLR showed a statistically significant but very weak positive correlation with stage IV NSCLC ( *r* = 0.198, *p* = 0.013), indicating limited explanatory value.

**Conclusion:**

The inflammatory indices were not significantly associated with disease stage or ^18^ F-FDG PET/CT SUVmax. Although NLR showed a statistically significant positive correlation with stage IV NSCLC, the effect size was very small and should not be interpreted as evidence of meaningful staging utility. Larger prospective studies are needed to determine whether readily available hematological indices such as SII, NLR, and PLR provide complementary value in prognostic or multimarker models in lung cancer.

## Introduction

Lung cancer is one of the leading causes of cancer-related mortality worldwide. Non-small cell lung cancer (NSCLC) accounts for ∼85% of all lung cancers. 1 Fiberoptic bronchoscopy (FOB), which allows direct visualization of endobronchial lesions and tissue sampling, is a fundamental diagnostic procedure in appropriately selected patients. Common indications include suspected malignancy, respiratory infection, and hemoptysis. 2 The clinical stage at diagnosis is a major determinant of prognosis and treatment selection because of the biological heterogeneity of lung cancer. ^18^ F-fluorodeoxyglucose positron emission tomography/computed tomography ( ^18^ F-FDG PET/CT) is widely used for staging and treatment planning. The maximum standardized uptake value (SUVmax) of the primary tumor reflects glucose uptake and may indirectly indicate biological aggressiveness. ^18^ F-FDG PET/CT is particularly sensitive for detecting bone and adrenal metastases. 3 Higher SUVmax values have also been associated with poorer clinical outcomes in some studies. In small cell lung cancer (SCLS), ^18^ F-FDG PET/CT may provide greater accuracy and sensitivity than conventional imaging for staging, response assessment, and prognostic evaluation. 4

The host systemic inflammatory response, together with microscopic and macroscopic tumor spread, may be associated with tumor progression, metastasis, and treatment response. Hemogram-derived inflammatory indices are inexpensive and readily available candidate biomarkers. The neutrophil-to-lymphocyte ratio (NLR) and platelet-to-lymphocyte ratio (PLR) have been associated with prognosis across different histological subtypes and treatment settings. Meta-analyses have shown that a high NLR is associated with worse survival and that a high PLR may indicate an unfavorable prognosis in NSCLC. 5 6 The systemic immune-inflammation index (SII), calculated as platelet count × neutrophil count / lymphocyte count, integrates three circulating cell types and has also been associated with worse overall survival in lung cancer. 7 However, the extent to which these markers reflect clinical stage and primary-tumor SUVmax among patients diagnosed by bronchoscopy remains unclear. Therefore, this study aimed to evaluate the associations of SII, NLR, and PLR with clinical stage and ^18^ F-FDG PET/CT SUVmax and to assess whether these simple hemogram-derived indices reflect disease extent or tumor metabolic activity.

## Materials and Methods

This retrospective study was conducted at the Chest Diseases Clinic of Evliya Çelebi Training and Research Hospital, Kütahya Health Sciences University Faculty of Medicine. Patients aged ≥18 years who underwent bronchoscopy and were subsequently diagnosed with lung cancer between January 1, 2023, and March 31, 2025, were eligible. Demographic characteristics, comorbidities, laboratory data, and ^18^ F-FDG PET/CT findings were extracted from the medical records; no personally identifiable information was collected. Complete blood count (CBC) values obtained at initial admission and the primary-tumor SUVmax from staging PET/CT were recorded. In all included patients, CBC parameters were obtained before any therapeutic intervention. ^18^ F-FDG PET/CT was performed as part of the routine pretreatment staging workup, with a median interval of 1 week after CBC acquisition. Clinical TNM stage was finalized after completion of imaging. Thus, hematological sampling and PET/CT imaging were performed during the same diagnostic period and before initiation of cancer-directed therapy.

All PET/CT examinations were performed using a GE Discovery 610 PET/CT system at our institution. Patients fasted for at least 6 hours before radiotracer administration, and blood glucose was confirmed to be ≤200 mg/dL. ^18^ F-FDG was administered intravenously at a weight-adjusted dose. After a standardized uptake period of 60 minutes in a quiet, temperature-controlled room, whole-body images were acquired from the skull base to the mid-thigh. Attenuation correction was performed using the CT dataset. SUVmax was defined as the maximum pixel activity within a three-dimensional volume of interest manually delineated over the primary lung lesion by an experienced nuclear medicine specialist and normalized to the injected dose and body weight. In patients with confirmed distant metastases, the SUVmax reported in this study corresponded exclusively to the primary tumor. NSCLC was staged according to the ninth edition of the TNM classification, 8 whereas patients with SCLC were classified separately.

The inclusion criteria were age ≥18 years, bronchoscopy performed because of suspected lung cancer, histopathological confirmation of lung cancer, and availability of complete staging and PET/CT data. Exclusion criteria were age <18 years, absence of a definitive lung cancer diagnosis after bronchoscopy, or unavailable retrospective PET/CT data. Because SCLC comprised a relatively small subgroup ( *n* = 28, 15.2%), the primary correlation analyses were restricted to patients with NSCLC ( *n* = 156) to improve biological homogeneity and analytical robustness.

### Statistical Analysis

Statistical analyses were performed using SPSS version 25. The distributional normality of numerical variables was assessed using histograms and the Kolmogorov-Smirnov or Shapiro-Wilk test, as appropriate. Numerical variables are summarized as mean ± standard deviation or median (interquartile range [IQR]), and categorical variables as number and percentage. Parametric or nonparametric tests were used according to the distribution of the data. Associations between categorical variables were evaluated using Pearson's chi-square test. Correlations were assessed using Pearson's or Spearman's correlation coefficient, as appropriate. NLR, PLR, and SII showed right-skewed distributions and are therefore presented as median (IQR). Between-stage comparisons of inflammatory indices were performed using the Kruskal–Wallis test. Correlations of NLR, PLR, and SII with disease stage and SUVmax were evaluated using Spearman's rank correlation coefficient. A two-sided *p* value <0.05 was considered statistically significant.

## Results

Of 357 patients who underwent FOB between January 2023 and March 2025, a total of 184 with complete staging data were included. Of these, 172 (93.5%) were male and 12 (6.5%) were female. Age ranged from 43 to 89 years, with a mean of 67.9 ± 8.4 years. Among the 175 patients with available smoking-status data, 10 (5.7%) were never-smokers, 52 (29.7%) were current smokers, and 113 (64.6%) were former smokers. Smoking exposure ranged from 5 to 120 pack-years, with a mean of 49.0 ± 21.5 pack-years. Mean tumor size was 56.3 ± 28.8 mm (range, 11–174 mm). Demographic and descriptive characteristics are presented in Table 1 .

**Table 1:** Demographic and descriptive information about the patients

	*n* (%)
Age (years), mean ± SD	67, 9 ± 8, 4
Gender	
Male	172 (%93, 5)
Female	12 (%6, 5)
Smoking history	
Non-smoker	10 (%5, 7)
Current smoker	52 (%29, 7)
Former smoker	113 (%64, 6)
Type of lung cancer	
NSCLC	156 (%84, 8)
SCLC	28 (%15, 2)
NSCLC stage	
1	14 (%7, 6)
2	23 (%12, 5)
3	53 (%28, 8)
4	66 (%35, 9)
SCLC stage	
Widespread	24 (%12, 0)
Localized	4 (%2, 2)
Metastasis	
Present	150 (%81, 5)
Absent	34 (%18, 5)
Endobronchial lesion on bronchoscopy	
Present	110 (%59, 8)
Absent	74 (%40, 2)
Mass lesion	
Central	136 (%73, 9)
Peripheral	48 (%36, 1)

Abbreviations: NSCLC, non-small cell lung cancer; SCLC, small cell lung cancer.

NSCLC was the most common histopathological diagnosis, accounting for 84.8% of cases. The mean ^18^ F-FDG PET/CT SUVmax for the overall cohort was 10.28 ± 5.59 (range, 1.7–31.30). The mean SUVmax was 10.37 ± 4.73 in patients with NSCLC and 9.71 ± 3.71 in patients with SCLC.

Because the primary analyses focused on patients with NSCLC ( *n* = 156), correlations between inflammatory indices and disease stage, as well as ^18^ F-FDG PET/CT SUVmax, were evaluated within this subgroup. Stage-specific median values are presented in Table 2 .

**Table 2:** Median (IQR) values of ^18^ F-FDG PET SUVmax values and biomarkers according to lung cancer stages

		Median (IQR)
^18^ F-FDG PET SUVmax	NSCLC stage 1	6, 67 (3, 05)
	NSCLC stage 2	10, 25 (3, 72)
	NSCLC stage 3	9, 25 (5, 37)
	NSCLC stage 4	10, 50 (6, 27)
NLR	NSCLC stage 1	4, 87 (9, 66)
	NSCLC stage 2	3, 34 (6, 22)
	NSCLC stage 3	4, 48 (5, 42)
	NSCLC stage 4	7, 38 (15, 97)
PLR	NSCLC stage 1	176, 18 (132, 73)
	NSCLC stage 2	203, 44 (181, 81)
	NSCLC stage 3	227, 18 (143, 39)
	NSCLC stage 4	195, 53 (281, 66)
SII	NSCLC stage 1	1227.010, 14 (2,687.822, 24)
	NSCLC stage 2	895.897, 14 (2,037.910, 16)
	NSCLC stage 3	1,497.535, 35 (1,789.790, 49)
	NSCLC stage 4	1,498.615, 39 (3,453.700, 44)

Abbreviations: NLR, neutrophil-to-lymphocyte ratio; NSCLC, non-small cell lung cancer; PLR, platelet-to-lymphocyte ratio; SII, systemic immune-inflammation index.

Neither SII, NLR, nor PLR showed a significant correlation with overall disease stage or ^18^ F-FDG PET/CT SUVmax. NLR showed a statistically significant but very weak positive correlation with stage IV NSCLC (Spearman's *r* = 0.198, *p* = 0.013). Given the small magnitude of the coefficient, this association has limited explanatory value and should be interpreted cautiously. Correlation results are presented in Table 3 .

**Table 3:** Correlations between disease stages and ^18^ F-FDG PET SUVmax values with NLR, PLR, SII

	NLR		PLR		SII	
	*p*	*r*	*p*	*r*	*p*	*r*
^18^ F-FDG PET SUV max	0.941	0.006	0.543	−0.050	0.907	0.010
NSCLC stage 1	0.357	−0.074	0.695	−0.032	0.584	−0.044
NSCLC stage 2	0.086	−0.138	0.561	−0.047	0.126	−0.123
NSCLC stage 3	0.478	−0.057	0.513	0.053	0.461	0.060
NSCLC stage 4	0.013 a	0.198	0.986	0.001	0.479	0.057

Abbreviations: NLR, neutrophil-to-lymphocyte ratio; NSCLC, non-small cell lung cancer; PLR, platelet-to-lymphocyte ratio; SII, systemic immune-inflammation index.

a Spearman's rank correlation.

## Discussion

In this study, we evaluated the associations of ^18^ F-FDG PET/CT SUVmax and lung cancer stage with NLR, PLR, and SII in 184 patients diagnosed by bronchoscopy. NSCLC was the predominant histological category. Although both NSCLC and SCLC were included, these entities differ substantially in biological behavior, staging, metabolic characteristics, and systemic inflammatory profiles. Because SCLC accounted for only 28 patients (15.2%), the primary correlation analyses were restricted to the NSCLC subgroup to improve biological homogeneity and analytical robustness; SCLC findings are presented descriptively. As shown in Table 2 , the highest median SUVmax was observed in stage IV NSCLC (10.50 [IQR, 6.27]). No significant correlations were found between SII, NLR, or PLR and overall disease stage or SUVmax. Although NLR was statistically correlated with stage IV NSCLC, the correlation was very weak ( *r* = 0.198) and therefore has limited explanatory and clinical value. This finding should not be interpreted as evidence that NLR can reliably identify or explain stage IV disease.

Lung cancer remains a leading cause of cancer-related death in both women and men, and NSCLC accounts for ∼85% of all cases. 1 The NSCLC rate in our cohort was 84.8%, consistent with the literature. Most previous studies have evaluated NLR, PLR, and SII in relation to survival rather than their direct correlation with anatomical stage. In a meta-analysis of patients with NSCLC receiving systemic therapy, a high pretreatment NLR was associated with worse progression-free and overall survival. 9 Similarly, a high PLR has been associated with worse survival in patients with lung cancer receiving immunotherapy, 10 and SII has shown prognostic value in SCLC and in lung cancer treated with immune checkpoint inhibitors. 11 12 Accordingly, our findings suggest that these markers may be more informative in prognostic or multimarker models than as standalone indicators of anatomical stage or tumor metabolic activity.

The reported relationship between ^18^ F-FDG PET/CT parameters and peripheral inflammatory markers has been heterogeneous. In patients with advanced NSCLC receiving first-line pembrolizumab plus platinum-based chemotherapy, combining metabolic imaging parameters with peripheral inflammatory markers improved prognostic stratification. 13 Another study showed that high SUVmax, a low lymphocyte-to-monocyte ratio, and a composite score incorporating both were associated with poorer prognosis in patients with EGFR-mutant stage IIIB-IV lung adenocarcinoma receiving chemotherapy. 14 More recently, inflammatory and nutritional indices evaluated in relation to metabolic tumor activity were associated with clinical outcomes in NSCLC treated with immunotherapy; these indices were higher in patients with overall survival of less than 1 year. 15 Collectively, these findings suggest that integrated models may be more clinically informative than any single biomarker. In the present study, however, no significant association was found between inflammatory indices and primary-tumor SUVmax.

Several factors may explain differences between our findings and those of previous studies. First, this study had a retrospective cross-sectional design, and the primary endpoint was the association of inflammatory indices with stage and SUVmax rather than survival. Second, the marked male predominance and the large proportion of patients with stage IV or extensive disease may have restricted variability and reduced statistical power. Third, inflammatory indices were measured at a single time point, and multivariable adjustment for potential confounders, including infection, inflammatory comorbidities, corticosteroid use, and performance status, could not be performed. Fourth, the imbalance between the SCLC and NSCLC subgroups may have weakened the observed inflammation-metabolism relationship. Fifth, SUVmax is a single-voxel measurement and may not adequately represent whole-tumor metabolic burden. Volumetric PET parameters, including metabolic tumor volume (MTV) and total lesion glycolysis (TLG), were unavailable because they were not systematically reported in routine PET/CT examinations and retrospective DICOM-based segmentation was not feasible. Sixth, although all patients were treatment-naive at CBC sampling, short-term antibiotic or corticosteroid exposure during the prediagnostic period was not documented uniformly. Consequently, the statistically significant NLR-stage IV correlation ( *r* = 0.198) should be interpreted cautiously: its very small magnitude indicates limited explanatory value, and residual confounding cannot be excluded. The study also has strengths, including bronchoscopic histopathological confirmation, real-world data, assessment of PET/CT and hematological markers during the same diagnostic period, and a relatively large single-center cohort. These features support the feasibility of evaluating inflammatory indices in patients undergoing bronchoscopy, although they do not establish clinical staging utility.

Future studies should use prospective multicenter designs, stratify analyses by histological subtype, obtain serial hemogram measurements, and apply multivariable models incorporating stage, performance status, treatment, and comorbidity. In addition to SUVmax, volumetric ^18^ F-FDG PET/CT parameters such as MTV and TLG should be evaluated with SII, NLR, and PLR to characterize more comprehensively the relationship among tumor metabolic burden, systemic inflammation, and clinical outcomes.

## Conclusion

Among patients with lung cancer diagnosed by bronchoscopy, SII, NLR, and PLR were not significantly associated with disease stage or primary-tumor ^18^ F-FDG PET/CT SUVmax. NLR showed a statistically significant but very weak positive correlation with stage IV NSCLC ( *r* = 0.198), a finding with limited explanatory value that should not be overinterpreted. These indices should not be used as standalone staging markers; their potential role may lie in complementary prognostic or multimarker models. Prospective multicenter studies with longitudinal sampling and multivariable adjustment are required.
